# Gender differences in the association between socioeconomic status and hypertension in France: A cross-sectional analysis of the CONSTANCES cohort

**DOI:** 10.1371/journal.pone.0231878

**Published:** 2020-04-20

**Authors:** Lola Neufcourt, Séverine Deguen, Sahar Bayat, Marie Zins, Olivier Grimaud

**Affiliations:** 1 Univ Rennes, EHESP, REPERES (Recherche en pharmaco-épidémiologie et recours aux soins)–EA, Rennes, France; 2 EHESP, IPLESP (Institut Pierre-Louis d’Epidémiologie et de Santé Publique) UMR S 1136, Rennes, France; 3 INSERM, Population-Based Epidemiological Cohorts Unit, UMS 011, Villejuif, France; 4 Paris Descartes University, Paris, France; Graduate School of Public Health and Health Policy, City University of New York, UNITED STATES

## Abstract

**Background:**

Hypertension prevalence increases when socioeconomic status decreases but gender differences in the relationship between socioeconomic status and hypertension have been less studied. This work aimed to explore the pattern of associations between three indicators of socioeconomic status at individual, household, and municipal levels with hypertension across genders in a large sample of French adults from the CONSTANCES cohort.

**Methods:**

Using data at inclusion from 59 805 participants (52% women) aged 25–69 years and recruited between 2012 and 2015, multilevel log-Poisson regressions with robust variance estimates were used to assess the associations of Relative Index of Inequality in education, monthly income per consumption unit and residential deprivation with hypertension. Modifying effects of gender and age in those associations were tested.

**Results:**

Hypertension prevalence was higher in men than in women. Steep socioeconomic gradients of hypertension were observed for the three socioeconomic indicators in both genders and from the youngest to the oldest age class. Socioeconomic inequalities, especially educational inequalities, were larger among women than men: Relative Index of Inequality for highest versus lowest education among the 25–34 years were 0.43 [95%-confidence interval = 0.28–0.67] in women and 0.70 [95%-confidence interval = 0.53–0.92] in men. With increasing age, socioeconomic gradients of hypertension eased in men and even more in women so that gender differences decreased.

**Conclusions:**

In this cross-sectional analysis of a large sample of adults, prevalence of hypertension was higher in men than in women. Moreover, socioeconomic status and especially education displayed a stronger association with hypertension prevalence in women compared to men. Reducing inequalities in hypertension may require gender-specific approaches.

## Introduction

Hypertension is a prominent risk factor for global burden of disease and especially for cardiovascular diseases which is the leading cause of death in the world (17.8 million [95% uncertainty interval 17.5–18.0] deaths in 2017) [1]. In 2010, hypertension was responsible for 51% and 45% of deaths due to stroke and coronary heart disease, respectively [2]. Its prevalence is increasing worldwide and reached 31.1% [95%-confidence interval (CI) = 30.0%–32.2%] in 2010 [3]. In France, hypertension prevalence is stable around 31% since 2006 [4]. Hypertension prevalence tends to be higher in men than in women, especially at young ages [5]. Indeed, higher age-standardized rate of hypertension in men have been reported in developed countries such as Canada, England or Australia but also in low- and middle-income countries like Mexico or Cameroon [6]. In France, a recent study conducted among participants from the CONSTANCES cohort (2012–2015) showed a 14-percentage point difference in hypertension prevalence between men and women (37% versus 23% respectively) [7].

Several determinants of hypertension have been identified including age, obesity, physical inactivity, excess sodium intake [8] and more recently socioeconomic status (SES) [9,10]. Socioeconomic inequalities in hypertension have been widely described and several indicators of SES measured at various levels have been associated with hypertension. In high-income countries, it is now well established that SES is inversely related to hypertension with greater risks generally observed among individuals with a lower level of education, a lower income and for those living in more disadvantaged areas [9]. In France, a lower educational level in particular has been associated with higher prevalence of hypertension [11,12]. As for other risk factors, social inequalities in hypertension are a major public health issue and reducing inequalities is one of the 17 Sustainable Development Goals defined by the United Nations Member States in 2015 [13]. However, whether associations between SES and hypertension differ between men and women is not clear, and to date, only few studies have investigated gender differences in these associations, most of them having been conducted in non-European countries [14–19]. Exploring gender differences and highlighting specificities or similarities between men and women could help understand mechanisms underlying socioeconomic disparities in hypertension, identify effective targets to act on to close the gap between socioeconomic groups and improve the general health of the population [20].

In this context, our study aimed to examine the pattern of associations between three indicators reflecting different dimensions of the SES and operating at individual, household, and municipal levels and hypertension across genders in a large sample of French adults from the CONSTANCES cohort. We also investigated the modifying effect of age on the association between SES and hypertension prevalence.

## Materials and methods

### Population

The CONSTANCES cohort is a large community-based cohort designed to be nationally representative of the French population of salaried workers affiliated to the National Health Insurance Fund (more than 85% of the French population, farmers and self-employed workers are excluded) [21] and aged 18–69 years at inclusion. The source population is restricted to individuals living in one of the 16 collaborating departments distributed across the French metropolitan territory (**S1 Fig**). Briefly, eligible persons were selected from the national social security database according to a random sampling scheme stratified on age, gender, SES and region of France in order to be representative of the source population. They received at home an invitation and those who volunteered and gave their informed consent were invited to undergo a free health examination in one of the collaborating Health Screening Centers. The full health check-up included anthropometric, physiological and biological measurements. Participants also filled in a series of self-administered questionnaires on demographic characteristics, SES, lifestyle, and personal and familial medical history. The CONSTANCES recruitment started in 2012 and aimed to include 200,000 individuals. The present study is based on compiled and cleaned data for 81 217 participants who agreed to take part in CONSTANCES between 2012 and 2015. The 2012–2013 participation rate was 7.4% [22], which is comparable to that of other cohort studies based on voluntary participation, such as the UK Biobank (5.5%) [23]. Compared with nonparticipants, participants were more likely to be male, to be older than 40 years of age, to have high occupational grade, to be out of the labor force, to earn more than average income (2,400 euros or approximately US $2,800 per month), to have regular medical follow-up and to have no chronic health problems [22]. Similar comparisons were drawn between CONSTANCES’ participants and the general population. Sampling weights were computed to account for nonparticipation, however, we did not use them in these analyses as they were year-specific.

In the present study, we excluded 21 412 individuals out of the 81 217 participants recruited in the CONSTANCES cohort between February 2012 and December 2015. We considered that participants younger than 25 years old may have not reached their highest level of education yet. The main reason for exclusion was a lack of matching with the national reimbursement database (N = 12 039 (56%)) (**S2 Fig**). Compared with excluded people, the 59 805 included participants were most often men and older (p<0.0001).

This study was approved by the National Data Protection Authority (Commission Nationale de l’Informatique et des Libertés—CNIL, number DE-2016-189), the Institutional Review Board (IRB00003888) and the CONSTANCES’ scientific committee. Data are accessible providing authorizations from the previously mentioned authorities.

### Blood pressure

Hypertension was defined as blood pressure measurements higher than 140/90 mmHg during the health check-up [24] and/or taking antihypertensive drugs.

Baseline blood pressure was assessed at inclusion by trained staff. A Standardized Operating Procedure [25] (available at www.constances.fr) and yearly inspected and calibrated oscillometric automated devices (OMRON® 705CP-II or OMRON® 705IT) with an appropriately sized cuff were provided by the CONSTANCES study coordinators. Blood pressure was measured three times after five-minute rest in lying position. The first measurement was taken on the right arm, the second on the left arm after 1min interval, and the third one on the arm with the highest value (reference arm) after another 1min interval. The mean of the two measurements on the reference arm was used as systolic and diastolic blood pressure. Only participants with three valid measurements were included in the present analysis.

To determine the presence of an antihypertensive treatment, the participants’ records were matched with the national health insurance reimbursement database to obtain an exhaustive history of their healthcare consumption before inclusion. This has proved to be a valid source of information for identifying cardiovascular treatments in epidemiological investigations [26]. Participants with at least one delivery of antihypertensive medication in a period of six months before inclusion were considered as treated for hypertension.

### Socioeconomic status

Because SES is a multidimensional construct, we included three complementary indicators, all expressed on an ordinal scale: self-reported education at an individual level, monthly income per consumption unit at the household level and deprivation index FDep at a municipal level. We chose to use education as an individual-level SES variable reflecting participant’s inner resources that is established at age 25 and easily comparable between countries. Household income was considered as a proxy for household material resources, which have been shown to be associated with cardiovascular health [27]. Although subject to under-reporting, this variable is fairly well completed in our sample with more than 92% of usable responses. Finally, the FDep index is a contextual SES variable that influences health on top and above individual characteristics. This is a composite score built at the municipality level using data from the 2009 French census which constitutes a relevant and validated tool widely used to assess neighborhood socioeconomic disparities in France [28]. Negative values of FDep index indicate low level of deprivation whereas positive values of FDep indicate high level of deprivation.

We divided education into five classes corresponding to specific attainments in the French context: up to lower secondary education (corresponding to up to 8 years of education), professional qualification (9–11 years), high school diploma or Baccalauréat (12–13 years), lower tertiary education (14–15 years), and higher tertiary education (16 years and more). We computed monthly income per consumption unit by dividing self-reported monthly household income by the number of consumption units per household, which was calculated according to the method recommended by the National Institute of Statistics and Economic Studies (INSEE): weighting 1 for the first adult in the household, 0.5 for other persons aged 14 years or older and 0.3 for children under 14 years) [29]. We categorized this indicator into quartiles. The FDep deprivation index measured at a municipal level [28] was also divided into quartiles.

### Covariates

Biological sex and age at inclusion were included as binary and categorical (five groups: 25–34; 35–44; 45–54; 55–64; more than 65 years of age) covariates, respectively.

### Statistical analyses

In descriptive analyses, continuous variables are expressed as means ± standard deviations (SD), and categorical variables are expressed as numbers (percentage). Age-standardized prevalence of hypertension and 95%-CI was estimated in the whole sample and separately among men and women using the reference French population for 2015, INSEE. We used t-tests and χ^2^ tests to compare respectively means and proportions between men and women. No sample weights were used in the presented analyses.

We examined the pattern of associations between SES and hypertension prevalence across genders using the Relative Index of Inequality (RII) [30]. The RII is a regression-based index that summarizes the magnitude of inequalities between SES groups. It takes into account both the size of the population and the relative disadvantage experienced by different groups. Individuals were ranked cumulatively from 0 to 1 according to their SES (education groups or quartiles of income per consumption unit and residential deprivation) so that “0” represented the lowest SES and “1”, the highest SES in the hierarchy. Each category was assigned a value based on the midpoint of the range in the cumulative distribution of the population of participants in the given category. Values of RII lower than 1 indicate that individuals with lower SES are more likely to experience hypertension compared with those with higher SES.

#### Statistical modeling

Literature suggests a growing preference for avoiding the use of odds ratios that are difficult to interpret [31]. Given the large prevalence of the hypertensive condition preventing the rare-disease assumption from being met and convergence issues related to log-binomial models, the RII in hypertension prevalence was calculated using multilevel log-Poisson regressions (integration method: mean-variance adaptive Gauss–Hermite quadrature) with robust variance estimations for the three socioeconomic indicators separately [32]. Multilevel modelling was justified by the design of the CONSTANCES cohort in which individuals (level 1) are nested within municipalities (level 2, median number of 3 participants per municipality) grouped by healthcare centres (level 3, median number of 3337 participants per center).

In order to investigate gender differences in the associations between each of the socioeconomic indicators separately and hypertension prevalence, we introduced an interaction term between RII and gender in the models. We also tested whether those associations differed by age and included an interaction term between RII and age group in gender-specific models. Thus, one model testing interactions and the corresponding stratified models (two for gender and five for age) were computed for each of the three socioeconomic indicators. In this paper, we present gender- and age-stratified models for education, income per consumption unit and residential deprivation. Fixed effects included socioeconomic status as well as gender and age, as appropriate. Random effects included municipalities and healthcare centres.

All statistical analyses were carried out with Stata, version 13 (StataCorp, United States).

#### Missing data

To avoid a reduction in sample size and bias related to listwise deletion in our models, the small fraction (<3%) of missing values for socioeconomic characteristics, namely education and income per consumption unit, was imputed by the multiple imputation (MI) procedure. The proportion of respondents in the non-imputed and imputed datasets is presented in **S1 Table**. We excluded from our analyses participants with non-informative variables (namely “other” for education and “don’t know/want to answer” for income).

## Results

**Table 1** shows the participants’ characteristics. Mean age was 48.9±12.6 years, and mean systolic and diastolic blood pressure values were respectively equal to 130±17 and 77±10 mmHg. The age-standardized prevalence of hypertension was 32.7% [95%-CI = 32.3–33.2], and was higher in men (40.2% [95%-CI = 39.4–40.9]) than in women (25.7% [95%-CI = 25.1–26.2], p<0.0001). Most participants reported higher tertiary education and median income per consumption unit was 1944€ per month, higher in men than women (p<0.0001). We imputed 1962 (3.3%) participants with missing data for education and/or income per consumption unit. The corresponding distributions were similar between the non-imputed and imputed datasets.

**Table 1:** Main characteristics of the study participants recruited in the French CONSTANCES cohort between 2012 and 2015, by gender (N = 59 805).

	All	Men	Women	P^b^
	N = 59 805	N = 28 664	N = 31 141	
Age, mean (SD)	48.9 (12.6)	49.5 (12.6)	48.3 (12.5)	< .0001
Systolic blood pressure (mmHg), mean (SD)	130 (17)	136 (15)	125 (16)	< .0001
Diastolic blood pressure (mmHg), mean (SD)	77 (10)	79 (10)	75 (9)	< .0001
Hypertension prevalence, % [95%-CI] ^a^	32.7 [32.3–33.2]	40.2 [39.4–40.9]	25.7 [25.1–26.2]	< .0001
Education, %				
Lower secondary education	9.4	9.2	9.6	< .0001
Professional qualification	17.7	21.5	14.2	
High school diploma (Baccalauréat)	15.1	14.3	15.9	
Lower tertiary education	24.5	20.5	28.2	
Higher tertiary education	31.6	32.8	30.5	
Other	0.2	0.2	0.2	
*Missing*	*1*.*5*	*1*.*6*	*1*.*4*	
Monthly household income per unit consumption (euros), median (IQR)	1944 (1139)	1963 (1486)	1944 (1163)	< .0001
Available, %	92.2	92.9	91.6	
Don’t know/ don’t want to answer, %	4.8	4.2	5.3	
*Missing*, *%*	*3*.*0*	*2*.*9*	*3*.*1*	
FDep index, median (IQR)	-0.643 (1.99)	-0.643 (1.98)	-0.643 (1.96)	0.61

CI: confidence interval; IQR: interquartile range; SD: standard deviation

^a^ Age-standardized prevalence using the 2015 French population

^b^ Chi-squared or Student’s t-test as appropriate (comparison between men and women)

**Fig 1:**
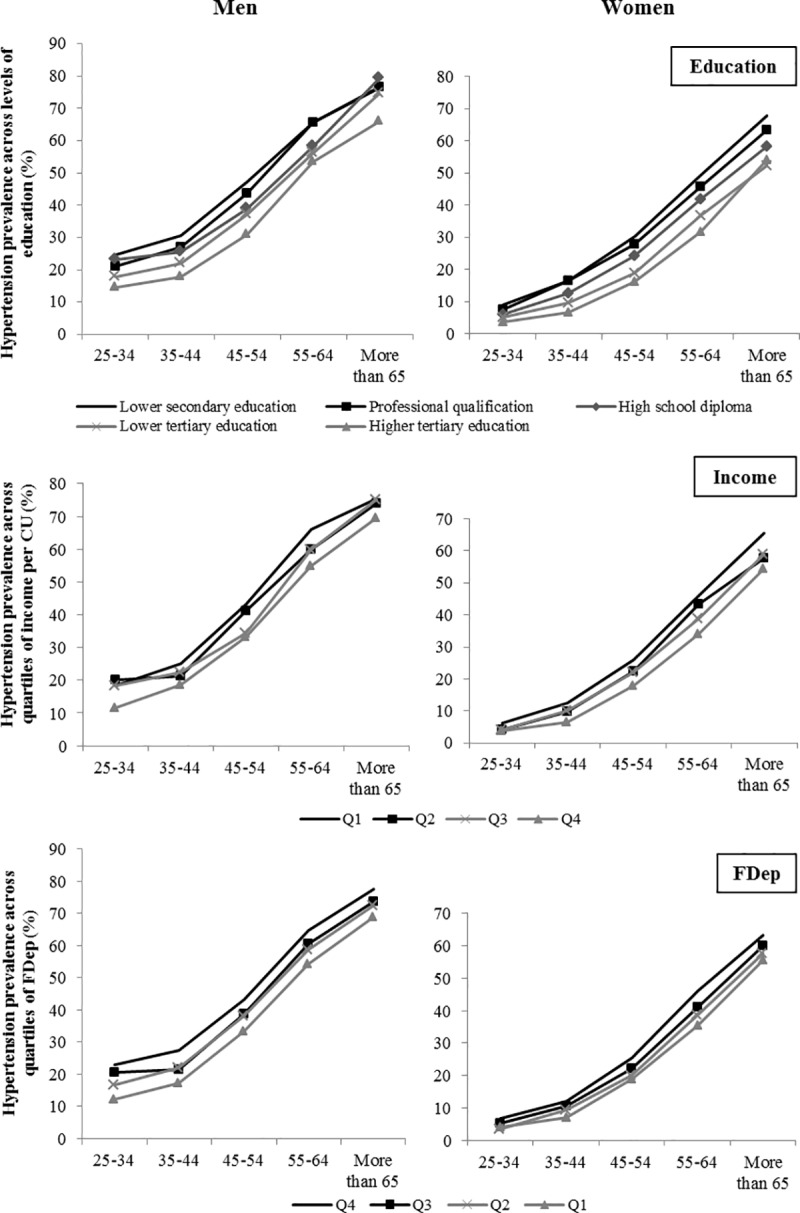
Socioeconomic variations in hypertension prevalence by gender and age groups, CONSTANCES cohort, 2012–2015 (N = 59 805). CU: consumption unit.

We found steep inverse socioeconomic gradients of hypertension: crude prevalence of hypertension was higher among participants with a lower level of education, a lower level of income per consumption unit or living in more deprived municipalities and gradually decreased as SES increased (**Fig 1**). Socioeconomic disparities in hypertension prevalence tended to be higher in women compared to men. There was a 5.4-percentage point absolute difference in hypertension prevalence between women with lower secondary education and women with higher tertiary education among the 25–34 years. The corresponding figure for men was 10.1-percentage point.

**Fig 2:**
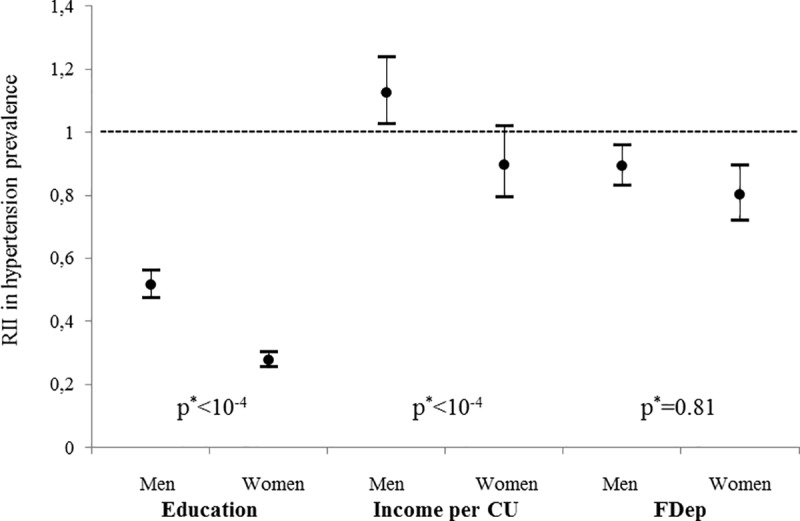
Relative index of inequality in hypertension prevalence among men and women for education, income and residential deprivation, CONSTANCES cohort, 2012–2015 (N = 59 805). CU: consumption unit; RII: relative index of inequality. RII compare the highest versus lowest level of education, the highest versus lowest quartile of income per consumption unit and the lowest (least deprived) versus highest quartile of FDep. ^a^ p for interaction between RII and gender.

There was a strongly significant interaction of gender in the association between education as well as monthly income per consumption unit and hypertension (p<10^−4^) but not for residential deprivation (p = 0.81). Gender-specific RII in hypertension prevalence are presented on **Fig 2** for the three socioeconomic variables. The largest disparities were observed for education, especially among women: those at the top of the educational hierarchy were more than three times less likely to have hypertension compared to those at the bottom (RII = 0.28 [95%-CI = 0.25–0.30]). The corresponding figure for men was RII = 0.51 [95%-CI = 0.47–0.56]). Individuals living in the least deprived municipalities were also less likely to have hypertension compared to those living in the most deprived areas and disparities were even stronger in women (RII = 0.80 [95%-CI = 0.72–0.89]) than in men (RII = 0.89 [95%-CI = 0.83–0.96]). Relative income inequalities in hypertension were less marked. We observed an inverse gradient of inequalities in men, which disappeared after adjusting on age groups.

**Fig 3:**
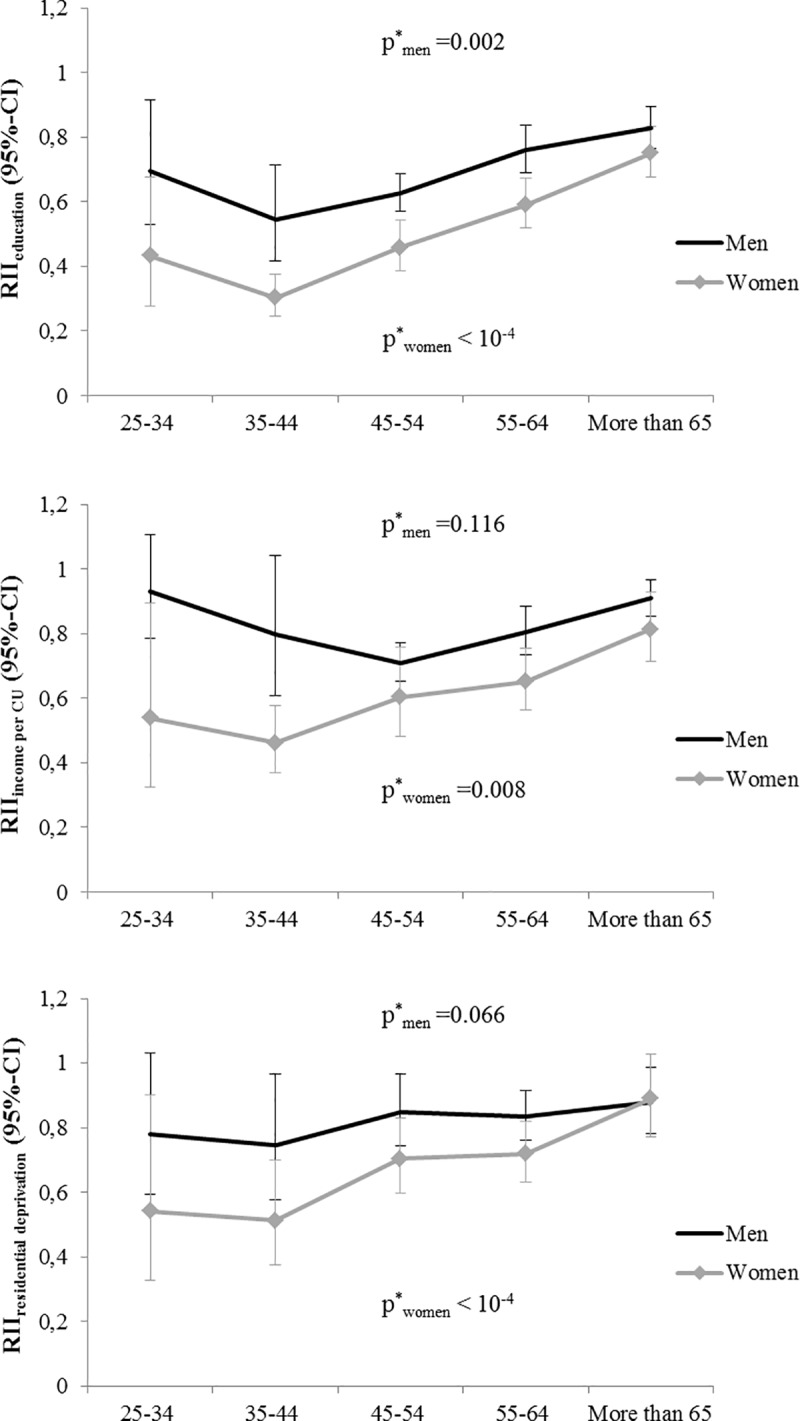
Relative index of inequality in hypertension prevalence among men and women across age groups, CONSTANCES cohort, 2012–2015 (N = 59 805). CI: confidence interval; CU: consumption unit; RII: relative index of inequality. RII compare the highest versus lowest level of education, the highest versus lowest quartile of income per consumption unit and the lowest (least deprived) versus highest quartile of FDep. ^a^ p for interaction between RII and age group.

We found a significant modifying effect of age in associations between RII for the three SES characteristics and hypertension in women and for education in men. Age-stratified models are presented on **Fig 3** separately for men and women. Higher education, higher income per consumption unit and lower residential deprivation (lower FDep) were associated with lower hypertension prevalence in men and women across all age groups. However, inequalities were larger among women compared to men for the three indicators. Moreover, differences between genders in the relationship of socioeconomic inequalities and hypertension prevalence across age groups varied depending on the SES indicator: they were marked and significant for education whereas they were less pronounced for income per consumption unit and absent for FDep. Looking at variations across age groups, relative socioeconomic inequalities globally tended to decrease in older age groups, and this was especially visible for education and residential deprivation. This diminution appeared to be stronger in women so that gender differences also tended to reduce in older age groups for all the three indicators.

## Discussion

This study contributes to the scarce literature investigating gender differences in the association between SES and hypertension prevalence. We found steep gradients of education, income per consumption unit and residential deprivation with hypertension prevalence in both genders. However, patterns of associations differed between genders with much stronger socioeconomic inequalities, especially educational inequalities, among women compared to men. These associations also varied across age groups and relative socioeconomic inequalities of hypertension between highest and lowest SES tended to decrease among older age groups. We observed a crude higher rate of hypertension among richer compared to poorer men, which is explained by a confounding effect of age.

Our work supports previous studies regarding the strong and inverse association between SES and hypertension prevalence [9,10,33]. Regarding gender differences, relative socioeconomic inequalities in hypertension were more prominent in women, suggesting that underlying mechanisms may differ between men and women. This has been reported previously in a few other studies that have investigated gender differences in socioeconomic disparities of hypertension [14,16,18,19]. However, most of them were conducted in middle-income countries. While the reasons for the gender-related differences remain unclear, several plausible explanations that relate low SES to an increased risk of hypertension in women deserve consideration. First, the pattern of lifestyle behaviors associated with SES can affect differently men and women [34]. For example, effect modification by gender in the association between SES and obesity, which is a strong determinant of hypertension, was found in various countries including France, education-related inequalities in obesity being stronger in women than in men [35]. Moreover, the association between SES and obesity has been shown to vary across genders according to the SES indicator considered. Indeed, literature shows that in contrast to women, the risk of being obese is the same among poorer and richer men [36]. Second, lower SES is also related to less social support or integration, and health beneficial effects of being more socially integrated may differ between men and women [37]. The working environment and the susceptibility to work-related stress or psychosocial exposure may also be differently experienced by men and women [38]. An additional explanation for the observed differences between genders may be related to the access to health care services. Poorer access to healthcare, which is observed among more disadvantage population, may lead to a lower detection and treatment of hypertension and associated risk factors. This phenomena may contribute to a greater extent to the SES gradient in women, who on average have more frequent contacts with a general practitioner than men [39]. To illustrate, socioeconomic gradient of treated hypertension in our sample was larger in women compared to men, with a 15.4 versus 9.4-percentage point difference between extreme levels of education.

An interesting point to underline is the gradual reduction in gender differences observed from younger to older age groups. In the 25–34 and 35–44 groups, gender differences are the highest for all the three indicators and this contrast tends to disappear in older age groups, where gender differences are no longer significant for education and income per consumption unit. We know that hypertension prevalence increases with age and that hypertension prevalence is higher in men than women approximately until the latter approach menopause, with associated mechanisms including the role of the kidneys, the renin–angiotensin system, relaxin, and developmental programming [40]. Thus, if we consider the growth of hypertension prevalence with age as an “epidemic” phenomena, one possible explanation could be that our results align the evolution of hypertension prevalence in both genders whereas the dynamics differ between men and women: at younger ages, the absolute prevalence is low but already quite high among men thus relative socioeconomic differences between men and women are strongly visible. As age increases, prevalence also increases in women and tends to catch up with the prevalence rate in men and relative socioeconomic inequalities between genders tend to decrease. In other words, instead of comparing hypertension prevalence between men and women in the same age group, it may be more meaningful to compare, for example, prevalence in women aged 45–54 or 55–64 years to prevalence in men aged 35–44 years. This pattern is particularly clear for education, which is the only socioeconomic indicator measured at the individual level in this study.

In our analyses, relative socioeconomic inequalities were visible at all ages and from the youngest group (25–34 years). However, they differed in magnitude across age groups as illustrated by significant interaction tests, especially in women and tended to decrease across age groups. Although some studies have indicated similar health inequalities in all age groups or increasing over age [41,42], other studies have suggested that health inequalities decrease with increasing age [43]. Also, it is possible that SES is no more a major determinant of hypertension prevalence at older ages, probably due to the increasingly stronger effect of age [44]. However, due to the cross-sectional design of this study, our results have limited ability to support these explanations. A cohort effect, which is plausible given marked educational attainment across generations, may partly explain the results.

SES is a multidimensional construct and the variety of indicators that may be utilized to define SES illustrates the complexity of this concept [45,46]. Also, special attention should be paid to the fact that different SES variables yielded different results. Unlike other studies which mostly focus on a single variable or a single level (individual or contextual), we used three different indicators to characterize SES in our study: i) individual level of education which is a valid, easily measurable, and stable indicator established during young adulthood which rarely changes over adult life and thus less subject to reverse causality; ii) monthly income per consumption unit which is strongly age-dependent and captures larger socioeconomic circumstances of the respondent’s household at a given point in time; iii) composite FDep index at a municipal level to account for the neighborhood characteristics of the participant’s SES. Those three variables reflect different dimensions and allow a very detailed description of the SES. Moreover, studying relative versus absolute inequalities makes it easier to compare three indicators measured at different levels. In the present study, relative socioeconomic inequalities in hypertension and gender differences in inequalities were more pronounced when using an individual-level indicator (education), less pronounced when using an indicator derived from household SES (income per consumption unit) and finally not really visible when using a municipal-level indicator (FDep). This suggests that individual-level indicators are appropriate to quantify relative socioeconomic inequalities in hypertension in the French context. Nevertheless, the multiple dimensions of SES used in our work offer a panel of effective targets at different levels and at various stages in life in terms of prevention of socioeconomic disparities in hypertension. Education represents an early determinant of inequalities that primary prevention should focus on. In order to better disentangle the complex picture of social inequalities, it can be recommended that different SES variables should be used and compared in future studies.

### Strengths and limitations

The major strengths of this study include: i) the use of a large sample that allowed precise estimations of socioeconomic variations of hypertension prevalence in gender- and age-stratified models; ii) the collection of a variety of socioeconomic variables which capture different dimensions and allow a detailed characterization of SES; and iii) the robust definition of hypertensive participants using the standardized blood pressure measurements along with the ascertainment of hypertension treatments by linkage to the national reimbursement database. However, as for many epidemiological investigations, the voluntary participation in CONSTANCES, resulting in typically healthier, higher educated and more health conscious volunteers, limits the representativeness of the sample and makes it difficult to extrapolate findings to the general French population. However, regarding the relatively homogeneous SES in our sample (55% of the sample reported tertiary education), we might assume that socioeconomic inequalities in hypertension would be even stronger in the general population. In addition, the cross-sectional design of this study limits both the ability to distinguish between cohorts and age effects, and conclusions regarding causality.

### Conclusions

This is the first French study to investigate patterns of socioeconomic associations with hypertension across genders using three different indicators of SES in a large sample of adults. We found that prevalence of hypertension was higher in men than in women. This work also underlines and confirms the existence of strong SES gradients of hypertension in both genders. This calls for specific prevention programs designed for individuals with lower SES. In addition, relative socioeconomic inequalities and especially educational disparities in hypertension prevalence were stronger in women compared to men, and this was observed from the youngest age group (25–34 years). Thus, gender-specific prevention measures targeting young adults may be required to address socioeconomic inequalities in hypertension. Besides, further studies with a longitudinal design are needed to explore the causal pathways linking SES and hypertension and identify mediators of this association to better understand specific mechanisms operating in women and men.

## Supporting information

S1 Table Proportion of imputed missing values for socioeconomic characteristics. (DOCX)

S1 Fig Distribution of the 16 CONSTANCES recruitment centers in mainland France. (TIF)

S2 Fig Flowchart. (TIF)

## List of abbreviations

CI: Confidence interval
CNAMTS: Caisse Nationale d’Assurance Maladie pour les Travailleurs Salariés
RII: Relative index of inequality
SD: Standard deviation
SES: Socioeconomic status
