# Supplementary material for: Gender differences in the association between socioeconomic status and hypertension in France: A cross-sectional analysis of the CONSTANCES cohort
Source: PLoS One. 2020 Apr 20;15(4):e0231878. doi: 10.1371/journal.pone.0231878 (PMC7170232; doi:10.1371/journal.pone.0231878)
Supplement: S1 Table — (DOCX) [file pone.0231878.s001.docx]

**S1 Table**

|  | Non-imputed dataset, N | Missing, N (%) | Imputed dataset, N |
| --- | --- | --- | --- |
| Education | 58 921 | 884 (1.5) | 59 805 |
| Monthly income per consumption unit | 58 024 | 1781 (3.0) | 59 805 |
