# Supplementary figures and images for: Gender differences in the association between socioeconomic status and hypertension in France: A cross-sectional analysis of the CONSTANCES cohort
Source: PLoS One. 2020 Apr 20;15(4):e0231878. doi: 10.1371/journal.pone.0231878 (PMC7170232; doi:10.1371/journal.pone.0231878)

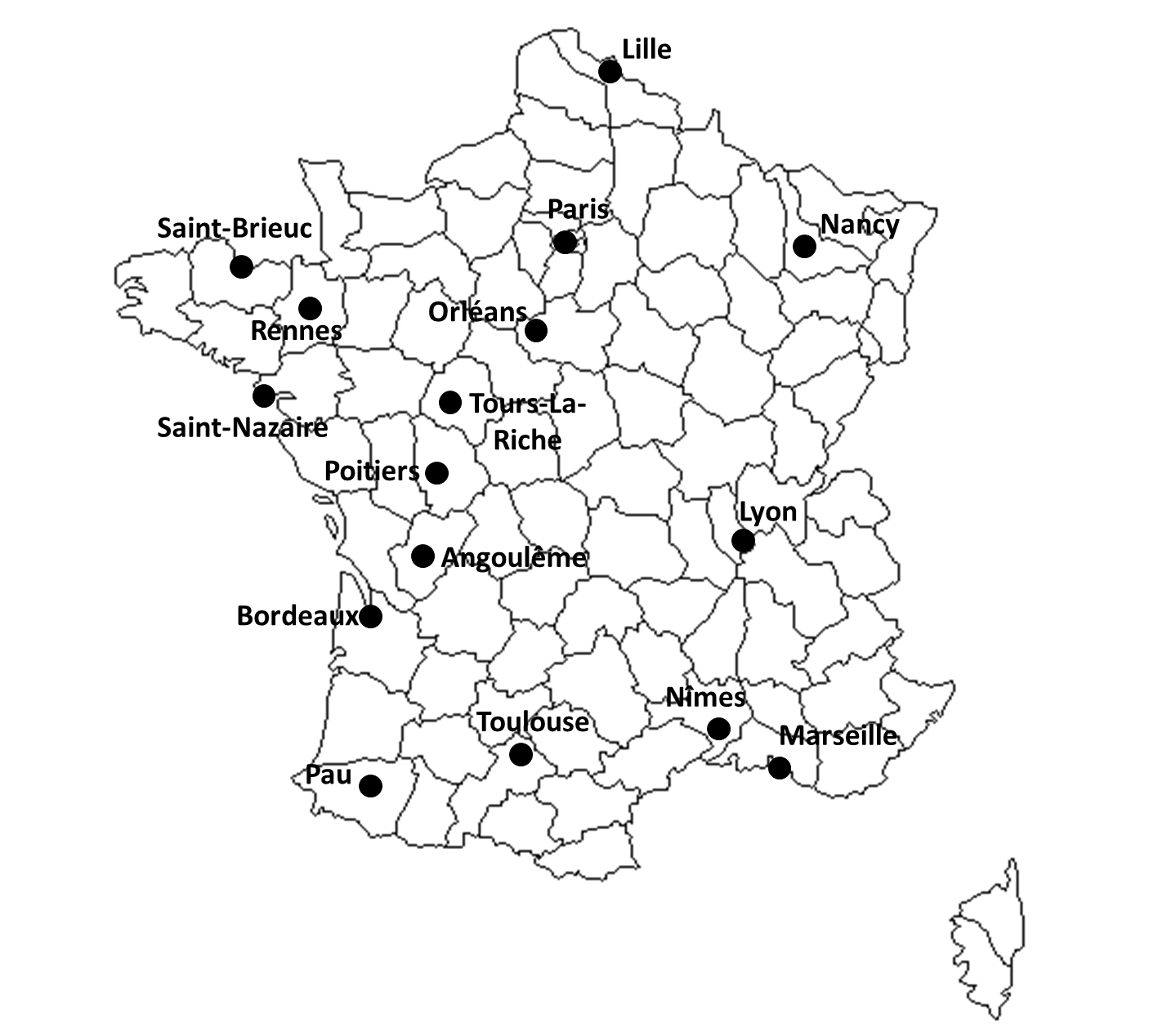

Supplement: S1 Fig — (TIF) [file pone.0231878.s002.tif]

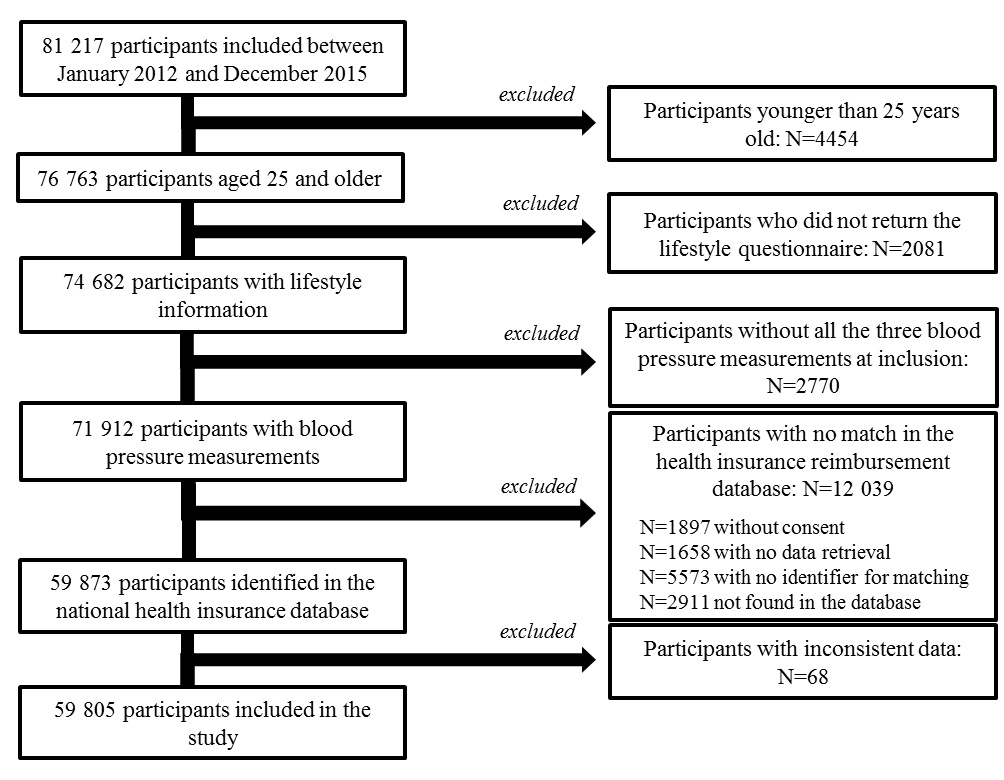

Supplement: S2 Fig — (TIF) [file pone.0231878.s003.tif]
